# African Primary Care Research: Performing a programme evaluation

**DOI:** 10.4102/phcfm.v6i1.634

**Published:** 2014-06-09

**Authors:** Lilian Dudley

**Affiliations:** 1 Community Health Division, Faculty of Medicine and Health Sciences, Stellenbosch University, South Africa

## Abstract

This article is part of a series on Primary Care Research in the African context and focuses on programme evaluation. Different types of programme evaluation are outlined: developmental, process, outcome and impact. Eight steps to follow in designing your programme evaluation are then described in some detail: engage stakeholders; establish what is known; describe the programme; define the evaluation and select a study design; define the indicators; plan and manage data collection and analysis; make judgements and recommendations; and disseminate the findings. Other articles in the series cover related topics such as writing your research proposal, performing a literature review, conducting surveys with questionnaires, qualitative interviewing and approaches to quantitative and qualitative data analysis.

## Abstrait

R**echerche africaine de soins primaires: Evaluation des Programmes**. Cet article fait partie d'une série sur la Recherche sur les soins primaires dans le contexte africain et se concentre sur l’évaluation des programmes. On y décrit différents types d’évaluation de programmes: développement, processus, résultats et impact. Les huit étapes à suivre pour organiser votre programme d’évaluation sont décrites en détail: nommer des parties prenantes; établir ce que l'on sait; décrire le programme; définir l’évaluation et choisir un plan d’étude; définir les indicateurs; planifier et gérer la collecte et l'analyse des données; faire des jugements et des recommandations; et disséminer les résultats. Les autres articles dans la série couvrent des sujets connexes comme la rédaction d'une proposition de recherche, la rédaction de revues d'articles, faire des enquêtes avec des questionnaires, des interviews qualitatives et les méthodes d'analyse de données quantitatives et qualitatives.

## Introduction

This article is part of a series on Primary Care Research and focuses on programme evaluation. This overview of programme evaluation provides a framework to guide health professionals and postgraduate health sciences students as to the type of programme evaluation that is relevant or required and the choice of research methods that would be appropriate in different contexts. The framework is based on the theory and practice of evaluation as described in scientific literature, which has been adapted for a postgraduate programme for health sciences students in South Africa. Some of the specific methods are described in detail in other articles in the series, such as surveys, questionnaires and qualitative interviewing, whilst others, such as randomised controlled trials, would require further reading and may not be feasible at a Masters level.

Evaluation is:

[*t*]he systematic collection of information about activities, characteristics, and outcomes of a program, services, policy or processes, in order to make judgements about the program/process, improve effectiveness, and/or inform decisions about future development.^1^

A wide range of health-related activities, including community mobilisation and communication campaigns, laboratory diagnostic services, training and education, direct service interventions, policy processes, surveillance systems and infrastructure programmes, can be evaluated. In this article, the term ‘programme evaluation’ will include evaluations of all such health-related interventions, processes and services. Further, it provides a framework for the performance of such evaluations, drawing on the theory and practice of evaluation in the literature and the experience and materials used in teaching evaluation methods for postgraduate health professionals at Stellenbosch University.

Evaluations are action oriented and inform judgements on whether a proposed programme should be started, how well an existing programme is functioning, or whether an established programme is achieving the desired effects. An evaluation therefore provides information so as to plan, correct, adapt and improve practices to enhance the likelihood of achieving the desired effects and to make decisions regarding the continuation, expansion or termination of a programme. An evaluation can also be used to build capacity, by enhancing the skills of health professionals and other stakeholders involved in a programme and by strengthening internal processes and accountability.

## Types of evaluation

Traditionally, evaluations were conducted at the end of the programme. Often this meant that problems, which arose during the planning and implementation, were not detected and corrected timeously. To be most beneficial and instrumental, evaluations should be conducted at all phases of the life of a programme.^2^

Evaluations during the planning of a new programme can ensure that what is being planned is appropriate. It clarifies whether it has merit and is acceptable to stakeholders affected by it. Thus, an evaluation can inform the conceptualisation of a programme by assessing the motivation for starting such a programme, define the need for the programme and inform decision makers about what the programme should look like. Such a *developmental evaluation* therefore assesses whether a new programme makes sense and assists in making decisions on whether or how it should be implemented.

An evaluation conducted during the implementation of a programme assesses whether the planned activities are indeed occurring and the intensity at which they are occurring. Such a *process or performance evaluation* also assesses the extent to which the selected target groups are being reached and whether the resources are available and being used efficiently in the delivery of the activities.

In well-established programmes, an *outcome or impact evaluation* provides evidence regarding whether the desired effects are being achieved. It answers the questions of whether the programme is meeting the stated goals and objectives, whether the target group or community has experienced any benefits or adverse effects and how effective it has been in bringing about the planned changes in healthcare or health status. Effects of a programme may be immediate, or may be longer term. Effects that occur soon after the programme delivery are generally described as ‘outcomes’ (e.g. changes in patient behaviour). Longer-term broader societal effects are described as ‘impacts’ (e.g. reduction in morbidity or mortality).^3^ It helps to distinguish the early outcomes from the longer-term impacts so as to identify the direct effects of the programme, which should be measured in the programme evaluation. An outcome evaluation could also include a costing of the resources used by conducting a cost analysis or a full economic evaluation to assess whether the benefits are worth the costs.^4^

A *comprehensive evaluation* includes all phases of a programme life cycle from the planning stages to the impact evaluation. Such a comprehensive approach ensures the alignment of the programme with the needs and desires of target groups and important stakeholders and continually informs adjustments to improve the delivery of the planned activities, increasing the likelihood of producing the desired outputs and achieving the planned outcomes and longer-term impact.

## Eight steps in conducting programme evaluation

The following eight steps are described as a guide for conducting a performance evaluation.

### Step 1: Engage stakeholders

Stakeholders are people with a vested interest, either as supporters or sceptics, who may potentially be affected by the programme and its evaluation.^2^ They could include community members, patients, health facility staff, health managers and other decision makers in the health system such as funders and politicians. Engaging stakeholders assists in the planning of the evaluation, by clarifying their interest and the extent to which they will either affect or use the evaluation.

A stakeholder mapping exercise is useful in identifying these individuals or groups and in categorising them into: (1) those involved in the delivery of the programme; (2) those served or affected by the programme; and (3) the intended users of evaluation findings.^2^ It is important to identify the interest in, perspective on and potential effect of the programme and the proposed evaluation on the different stakeholder groups, in order to select key stakeholders who should be engaged throughout the evaluation process.

### Step 2: Establish what is known

A well-designed evaluation should be informed by a critical review and synthesis of the literature, including local and contextual data.^5^ Evaluators should be apprised of current knowledge regarding the problem that the programme is seeking to address, best practices and the effectiveness of similar programmes and evaluations. Establishing a practice of evidence-informed processes in planning the evaluation will promote an approach of evidence-informed decisions throughout the evaluation.

### Step 3: Describe the programme

A clear programme description provides a frame of reference for all subsequent decisions, connects programme components to their effects and enables comparisons with similar programmes. The first step is to clarify the *stage of development of the programme* and the purpose of the evaluation. Will the evaluation be used to assist with planning during the conceptualisation of a new programme, to assess processes during implementation of a programme, to measure the effects of a mature programme, or will it be used for all stages of the programme? Then, clarify *the need for the programme* by defining clearly the problem that the programme seeks to address, identifying who the problem affects, as well as the causes of the problem. It is also important to understand how the need for the programme was determined and to assess whether the programme intent is appropriate. Describe the *context of the programme*, particularly the setting and local influences (e.g. geography, history, politics, social and economic conditions, as well as related activities of organisations) within which the programme operates.^6^ Understanding these influences can assist in the design of a context-sensitive evaluation, as well as in the interpretation and assessment of the generalisability of the results.

The *expected effects* of the programme should indicate what the programme should achieve in order to be considered successful. The mission, goals and objectives of the programme should be described in sufficient detail so as to ensure an understanding of the expected effects. Goals generally provide a broad statement of a desired, long-term outcome of the programme, whereas objectives are statements of desired, specific, realistic and measurable programme results. As different stakeholders may have different views of the goals and objectives of the programme, this should be clarified with the different groups at the outset of the evaluation.

The available *resources* such as the human resources, time, equipment, information, money and other assets available to conduct programme activities should also be ascertained.

**FIGURE 1:**
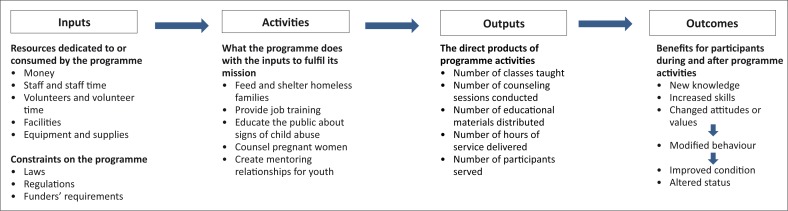
A programme logic model. *Source*: Hatry 1996^16^

Lastly, consider the programme's capacity to effect the desired change, based on the problem, the intervention, the context and resources. In short, why should this intervention work? A description of the ‘*programme design*’ or ‘*programme logic*’ assists by explaining why and how an intervention works. The logic model should clearly specify the resources required (inputs) for the programme, the activities that will be undertaken, the measurable products of the programme activities (outputs) and the benefits to clients, communities, systems or organisations (outcomes). The programme logic can be represented graphically through a ‘logic model’ which details the activities of a programme and the links between each phase, to assist in the planning, implementation, monitoring and evaluation of the programme (Figure 1). Logic models can be represented in different ways, including graphic displays of boxes and arrows, matrices in tables, or narratives which set out the logical arguments for a programme.^7^ Thus, the logic model assists in detailing and clarifying the relationship between different components of the programme, as well as assisting in an evaluation by identifying important components to be measured and appropriate indicators to be used at the different stages of the programme. The programme description and logic model's accuracy should be confirmed by consulting with stakeholders, or by direct observation of activities in the field.

### Step 4: Define the evaluation question and select a study design

The evaluation question determines the selection of the study design to be used for the evaluation. Without knowing what question(s) the evaluation is intending to answer, you cannot begin to consider methods or data sources. Engage the stakeholders in clarifying the questions of concern to decision makers through guided probes such as ‘what do you hope to know at the end of this evaluation that you don't know now?’, rather than ‘what are the evaluation questions?’.

Habicht presents two considerations for selecting an appropriate study design to address evaluation questions: firstly, the stage of the programme and secondly, the inferences to be made by the evaluation.^8^ In the programme planning stage, a developmental evaluation answers questions regarding whether the planned programme addresses the causes of the problem for the affected persons or groups, in a way which is likely to be effective and is acceptable and appropriate to the context. Whereas a process evaluation typically addresses questions such as whether services are available and accessible, of a suitable standard or quality and are being used (utilisation), as well as whether the target population is being reached (coverage). An outcome or impact evaluation answers questions regarding whether improvements in health-related behaviours, disease patterns and health status occurred. It therefore seeks to assess the effects of a programme, as well as its impact on stakeholders or participants.

The second important consideration relates to the type of inference to be made. Do decision makers simply want to know whether the programme goals and expected changes have been achieved, or do they want to establish whether the programme was the cause of the outcomes achieved? This differentiates between ‘contribution’ of the programme and direct ‘attribution’ of the effects to the programme. Is it important to establish a causal relationship; and how confident do you need to be that observed effects were as a result of the programme interventions?

The three types of evaluation based on the inferences and ‘level of certainty’ required from an evaluation are: (1) adequacy, (2) plausibility and (3) probability assessments. At the lowest level of inference and certainty, an *adequacy assessment* simply answers the question, ‘Did the expected changes occur?’, without establishing any causal relationship between the programme activities and the changes. An adequacy assessment can indicate if activities were performed as planned, whether objectives were met and if the expected changes occurred, without inferring that the observed changes were because of the programme. It can also indicate whether resources were utilised efficiently or not. A *plausibility assessment* establishes if there was some form of a causal relationship between the programme and the outcomes and answers the question, ‘Did the programme seem to have an effect above and beyond other external influences?’.^8^ A *probability assessment* evaluates whether there was a direct causal relationship between the programme and the outcomes, as well as the strength of this relationship. Such an assessment aims at ensuring that there is only a small known probability that the difference between programme and control groups is a result of confounding problems, bias, or chance.

Study designs for evaluation are drawn from scientific research methods, particularly those developed in the social, behavioural and health sciences.^1, 6^ A broad classification of design types includes experimental, quasi-experimental and observational designs. Of these, observational study designs, which do not require controls but may have before-and-after comparisons, are suitable for an adequacy assessment. Quasi-experimental designs are best suited for a plausibility assessment. They include before-and-after comparisons, have control groups and seek to identify and manage the effects of confounding factors. Such quasi-experimental study designs include before-and-after controlled studies, or time series analysis.^9^ Probability assessments require randomisation of intervention and control activities and thus require an experimental study design such as a randomised controlled trial.

Study designs should be selected during the programme planning, before implementation of the interventions, to allow for allocation of intervention and controls, including possible randomisation. Often this is not feasible, ethical or desirable, or does not occur for political or pragmatic reasons, so many evaluations of ‘effect’ are based ultimately on observational studies.^10^ Failure to plan the evaluation early, however, limits the choice of study designs and the ability of an evaluation to answer questions, which may be important for decision making.

In addition to ascertaining whether an intervention worked (and to measure accurately any difference it made), it may be important to understand why the intervention worked, including characteristics associated with success or failure, as well as the pathways through which effects are generated.^5^ The evaluation methods may thus require both quantitative approaches to measure effects, as well as qualitative approaches to understand why it worked. Such mixed-method approaches are able to address different evaluation questions and provide a comprehensive perspective of the programme.^11^

Lastly, in selecting a study design, consider the feasibility and ethical aspects of the proposed evaluation question and study design. How realistic are the proposed evaluation activities given the available time, resources and expertise? Is there adequate focus, or are you trying to do too much? Identify the ethical issues related to the context, participants and type of evaluation you are conducting in order to ensure that the evaluation is conducted ethically, legally, and considers the welfare of those involved and those affected.^12^ Approvals from research ethics committees or from institutions to access routine data, staff, facilities or internal reports may be required. As these applications take time to be processed, it is advisable to commence applications early in the planning of the evaluation.

### Step 5: Define the indicators

Having clarified the evaluation questions and study design, the next step is to select suitable indicators to measure the inputs, activities and outcomes of interest in the evaluation. Indicators are informed by the criteria that will be used to judge the programme. Thus, they should be meaningful with regard to addressing the evaluation questions and should align with and provide measures of the stated goals and objectives of the programme. Indicators can include measures of programme inputs (e.g. staff time, financial resources, materials and tools), activities (e.g. the participation rate, coverage rates and the efficiency of resource use) and measures of programme effects (e.g. changes in participant behaviour or practices, health status, quality of life or policies).

Characteristics of good indicators are that they:

[*s*]hould actually measure what they are intended to (validity); they should provide the same answer if measured by different people in similar circumstances (reliability); they should be able to measure change (sensitivity); and, they should reflect changes only in the situation concerned (specificity). In reality, these criteria are difficult to achieve, and indicators, at best, are indirect or partial measures of a complex situation.^13^

Multiple indicators may be needed for tracking the implementation and effects of a programme. The programme logic model (or other conceptual frameworks) provides a useful structured approach to defining key indicators leading from programme inputs to activities to expected effects.^7, 14^ By relating indicators to the logic model, the early detection of small changes in performance is possible, rather than if a single outcome were the only measure used.

### Step 6: Plan and manage data collection and analysis

The first step is to identify appropriate data sources for measurement of the indicators. Sources may include persons, documents or observations, which provide narrative (qualitative) or numeric (quantitative) data. Persons may provide information through surveys, interviews, focus group discussions or consensus development processes such as Delphi techniques. Documentary sources include routine health information systems, health service reports, grant proposals, press releases, meeting minutes, data records, presentations, articles, charts, photographs and videotapes. Routine health data sources are an important source of evaluation data as they are easily accessible, inexpensive and reflect directly on service activities and outputs. However, routine data are often incomplete or of poor quality and may need to be complemented by other sources such as survey data, for instance population census data, demographic or health surveys and facility surveys. Primary collection of new quantitative data may also be required for important indicators or to validate routine or survey data sources. Observations may include patient–provider interactions, service delivery, meetings, special events and other activities. The integration of data from different sources provides for a balanced evidence base, to respond to the needs and expectations of a diverse set of users.

A credible evaluation requires good quality data which are reliable, valid and appropriate for the intended use. Factors which affect the quality of the data collected include the indicator definitions, instrument design, data-collection procedures, training of data collectors, data source selection, coding, data management and routine error checking. Although all types of data have limitations, an evaluation's overall credibility can be improved by using multiple sources and procedures for gathering, analysing and interpreting data. A clear data collection and management plan assists by defining the processes, logistics and resources required for, as well as procedures to ensure quality control of, the data collection.

As part of the data management plan, describe how the data will be analysed in order to provide information to address the evaluation questions and to measure whether the programme has achieved its goals and objectives. Demonstrate how data sets, such as surveys or focus groups, will be analysed separately, as well as how the different sources of information will be combined or synthesised so as to reach a larger understanding of the programme performance. Mixed-method evaluations thus require the separate analysis of each element and a synthesis of different sources in order to examine patterns of agreement, convergence or complexity.^11^

Ultimately, the evidence generated by the evaluation needs to be credible to decision makers and other stakeholders. Credibility is affected by the extent to which stakeholders perceive it to be responsive to their needs and concerns; and by whether the study design is able to answer the evaluation questions. Other important factors include compliance with ethical practice, the quality of the data collected, the appropriateness and rigour of the data analysis and the approach used to interpret the results. Encouraging participation by stakeholders throughout the evaluation process can enhance perceived credibility. Stakeholders will be more likely to accept the evaluation's findings and to act on its recommendations, when they have been involved in defining, gathering and interpreting data that they find credible.^1^

### Step 7: Make judgements and recommendations

The main purpose of an evaluation is to inform judgements of programme merit, worth or significance and thereby to contribute to decisions and actions on continuation, expansion or termination of a programme. Such judgements are made by comparing the findings and interpretations of the evaluation to the stated goals and objectives of the programme and to selected standards determined with stakeholders. Involving stakeholders in processes to interpret the data, assists by drawing on their information and perspectives to inform the judgements made about the programme. This, in turn, enhances stakeholders’ perceptions regarding the credibility of the judgements and can influence the extent to which the evaluation will give effect to decisions and actions.

Recommendations on actions for consideration by decision makers should be based on the findings, their interpretation, the judgements made regarding programme performance and the context of the programme and its evaluation. Sharing draft recommendations, obtaining responses from multiple stakeholders and presenting options instead of directives ensures that recommendations will be relevant and well-received.

### Step 8: Disseminate the findings

Finally, dissemination of findings of the programme evaluation can facilitate their use to inform programme decisions and practices and further knowledge generation. Mechanisms for dissemination include reports to decision makers, presentations to health service, community and other forums, scientific publications, the use of electronic and other media.^15^

## Conclusion

These eight steps provide an introduction to important processes and considerations in performing a programme evaluation. However, they are not a comprehensive study of the theory and practice of evaluation methods and readers are encouraged to source additional resources when planning and conducting a programme evaluation.
